# Influence of Absorber Contents and Temperatures on the Dielectric Properties and Microwave Absorbing Performances of C@TiC/SiO_2_ Composites

**DOI:** 10.3390/nano14242033

**Published:** 2024-12-18

**Authors:** Yan Wang, Xin Sun, Zhihe Xiao, Jian Gu, Qinyi Dong, Shuhang Yi, Junyang Jin

**Affiliations:** 1 National Key Laboratory of Scattering and Radiation, Beijing 100854, China; sunxin52199@163.com (X.S.); xiaohe66479@vip.sina.com (Z.X.); dqy15203827961@126.com (Q.D.); 18811023822@163.com (S.Y.); 2 Beijing Institute of Environmental Features, Beijing 100854, China; 3 College of Materials Science and Engineering, Nanjing Tech University, Nanjing 211816, China; jjy@njtech.edu.cn; 4 Jiangsu Collaborative Innovation Center for Advanced Inorganic Function Composites, Nanjing Tech University, Nanjing 211816, China

**Keywords:** core–shell-structured C@TiC, C@TiC/SiO_2_ composites, high-temperature microwave absorption performance

## Abstract

TiC provides a promising potential for high-temperature microwave absorbers due to its unique combination of thermal stability, high electrical conductivity, and robust structural integrity. C@TiC/SiO_2_ composites were successfully fabricated using a simple blending and cold-pressing method. The effects of C@TiC’s absorbent content and temperature on the dielectric and microwave absorption properties of C@TiC/SiO_2_ composites were investigated. The addition of C@TiC from 10 wt.% to 30 wt.% not only endows the composites with a higher dielectric constant and dielectric loss, but also with a greater high-temperature stability in terms of dielectric and microwave absorption properties. The composite with 30 wt.%C@TiC demonstrates a strong microwave absorption capability with a minimum reflection loss (*RL_min_*) of −55.87 dB, −48.49 dB, and −40.36 dB at room temperature, 50 °C, and 100 °C, respectively; the 50 wt.%C@TiC composite exhibits an enhanced high-temperature microwave absorption performance with an *RL*_min_ of −16.13 dB and −15.72 dB at 200 °C and 300 °C, respectively. This study demonstrates that the TiC-based absorbers present an innovative solution for high-temperature microwave absorption, providing stability, versatility, and adaptability in extreme operational environments.

## 1. Introduction

Titanium carbide (TiC) has emerged as a promising material for high-temperature microwave absorbers due to its unique combination of thermal stability, high electrical conductivity, and robust structural integrity [1]. These properties make TiC particularly well suited for applications that demand consistent microwave absorption performance under extreme thermal conditions, such as in aerospace, military, and high-power electronic systems. Conventional microwave absorbing materials, often limited by their low melting points and susceptibility to oxidation, struggle to maintain efficiency in high-temperature environments. In contrast, TiC’s exceptional melting point (over 3000 °C) and resistance to oxidation make it highly durable, allowing it to function effectively in environments where traditional materials would fail.

TiC-based microwave absorbers leverage several intrinsic mechanisms for efficient microwave attenuation, including dielectric loss and conduction loss, driven by TiC’s free electron behavior and carbide bonds [2]. Additionally, its ability to absorb microwaves across a broad frequency range can be finely tuned by adjusting parameters such as particle size, morphology, and composite formulations. Previous research has shown that TiC’s performance can be further enhanced when combined with carbon-based materials, creating composites that exhibit improved impedance matching and broader absorption bandwidths. For instance, Yuan et al. [3] reported that TiC nanoparticle-decorated carbon spheres (C@TiC) exhibited an *RL*_min_ of −50.3 dB at 13.1 GHz with a thickness of 1.5 mm; with an increase in Ti, the absorption of C@TiC in paraffin gradually moved from the Ku to the S band. Wang et al. [4] fabricated core–shell-structured C@TiC nanocomposites by pyrolyzing the amorphous Ti-based metal–organic framework (aTi-MOF) precursor; the as-prepared C@TiC nanocomposites presented a high microwave absorption performance, including a stronger *RL* peak of −35.64 dB (10.72 GHz) at a 2.4 mm thickness, as well as an enhanced effective microwave absorption band width (EAB, *RL* ≤ −10 dB) that spans the entire C-band and X-band. Yuan et al. [5] reported that the addition of 7.5 wt.%TiC into SiO_2_ displayed a good microwave absorption performance in the temperature range from room temperature to 300 °C. Hence, it can be expected that the TiC-based absorbers present an innovative solution for high-temperature microwave absorption. Nevertheless, until now, very little work has been published on their high-temperature dielectric and microwave absorption properties.

In this work, the core–shell-structured C@TiC nanoparticles were synthesized by pyrolyzing the aTi-MOF precursor at 1500 °C under a vacuum atmosphere. In order to characterize the high-temperature microwave absorption performance of C@TiC absorbers, SiO_2_ is selected as the transparent matrix due to its high dielectric properties, chemical stability, and thermal resistance. Then, the C@TiC/SiO_2_ composites were fabricated using a simple blending, cold-pressing, and sintering method, in which C@TiC nanoparticles were used as electromagnetic (EM) wave absorbers and SiO_2_ was used as an EM wave transparent matrix. The dielectric properties and microwave absorption abilities were evaluated in the temperature range from room temperature to 400/800 °C at 8.2–12.4 GHz (X-band). Subsequently, to find the optimal reflection loss and the suitable thickness, quarter-wavelength plots were constructed. The microwave absorption mechanism is further discussed below.

## 2. Materials and Methods

Details about the synthesis of core–shell-structured C@TiC nanocomposites have been described elsewhere [4]. The fabrication process of waveguide composites involved initially mixing silica sol (30 wt.%) with ethanol (70 wt.%), followed by mechanically stirring the mixtures for half an hour. Then, various amounts of C@TiC nanoparticles were gradually added into the mixture of silica sol with ethanol while continuing to stir. After continuing to stir for 30 min, the mixtures were placed in an oven to dry at 80 °C for 5 h, until the solvent fully evaporated. The xerogel was ground into a powder using a mortar. Finally, the ground powders were molded into a rectangular shape with a pressure of 5 MPa and a dwell time of 10 min; the as-prepared C@TiC/SiO_2_ composites were sanded to the desired size (22.86 mm × 10.16 mm × 2 mm) using fine sandpaper.

The phase composition of the as-prepared C@TiC/SiO_2_ composites was identified using X-Ray diffraction (XRD; SmartLab, Rigaku, Tokyo, Japan). The microstructure and element distribution of the as-prepared composites was characterized using scanning electron microscopy (SEM; JEM-7600F, JEOL, Tokyo, Japan) equipped with energy-dispersive spectroscopy (EDS). Based on the transmission line technique [6], the complex permittivity and electromagnetic parameters of the C@TiC/SiO_2_ composites ranging from room temperature to 800 °C were determined using a vector network analyzer (N5234B PNA-L. KEYSIGHT, Santa Rosa, CA, USA) in the X band (8.2 to 12.4 GHz). Before testing, the waveguide samples were placed into a tube furnace, the temperature was set from room temperature to 400/800 °C, and the heating rate was maintained at 10 °C/min.

## 3. Results and Discussion

### 3.1. Phase Composition, Microstructure Characterization, and Thermal Stability

**Figure 1:**
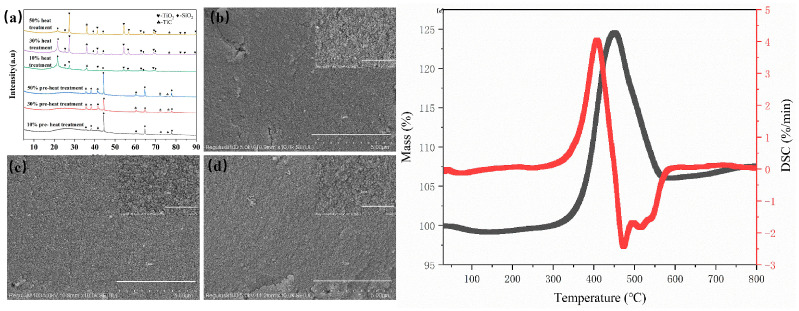
(**a**–**d**) XRD patterns and SEM images of the as-prepared C@TiC/SiO_2_ composites with different C@TiC contents. (**a**) XRD before and after high-temperature exposure; (**b**) 10 wt.%; (**c**) 30 wt.%; (**d**) 50 wt.%. The insets present enlarged aera. (**e**) TG-DSC curves of C@TiC nanoparticles.

The XRD patterns of the as-prepared composites are displayed in Figure 1a. As can be seen, no peaks were observed other than the SiO_2_ and TiC peaks. Notably, a broad peak is detected at the two Theta angle of ~25°, which is attributed to the low crystallinity of the carbon shell, indicating that the core–shell structure of C@TiC was preserved. Compared to the samples before high-temperature exposure, a new phase such as Titania appeared, which is caused by the TiC core partially oxidizing at 800 °C. Meanwhile, the carbon shell was oxidized into carbon dioxide gas. In addition, the crystal structure of SiO_2_ changed from α to β after high-temperature exposure. The microstructures of the as-prepared composites are presented in Figure 1b–d. With the increase in C@TiC content, no distinct difference was found; only nanostructured particles were observed for all cases. Figure 1e displays the thermal stability of the as-prepared core–shell C@TiC nanoparticles under an air atmosphere. As can be seen, no obvious mass change is observed at the temperature range from 30 to 372.3 °C. A severe mass variation occurred from 372.3 to 542.1 °C, whereby the weight gain initially increases up to 125.21% and then decreases down to 106.5%; this variation is attributed to the oxidation of the TiC core and carbon shell. Based on the ideal oxidation reaction of TiC, the theoretical weight gain should be 33.4%, which is higher than the measured value. This difference is caused by the simultaneous oxidation of the carbon shell and the carbon dioxide gas produced by the oxidation reactions, which decreases the weight gain. As the temperature increases, a small weight gain (1.55%) is observed, which is possibly provided from the further oxidation of the TiC core.

**Figure 2:**
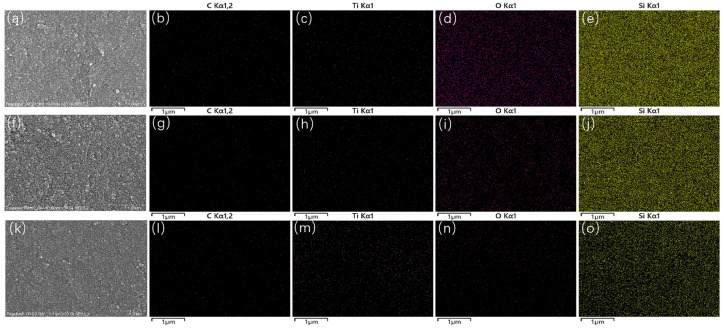
SEM images and mapping EDS results of the as-prepared C@TiC/SiO_2_ composites with different C@TiC contents. (**a**–**e**) 10 wt.%; (**f**–**j**) 30 wt.%; (**k**–**o**) 50 wt.%.

Figure 2 displays the mapping results of the as-prepared composites with various C@TiC absorber contents. It can be seen that only Ti, O, C, and Si elements are detected and uniformly distributed. With an increasing addition of C@TiC, the C and Ti elements shown in Figure 2 become more obvious. Conversely, the Si and O element distribution decreases. Moreover, according to the XRD results, the composition of the C@TiC absorbers and SiO_2_ transparent matrix can be determined. The uniform interfaces proved from the well-distributed C@TiC/SiO_2_ are conducive to the microwave absorption.

### 3.2. Dielectric Properties

**Figure 3:**
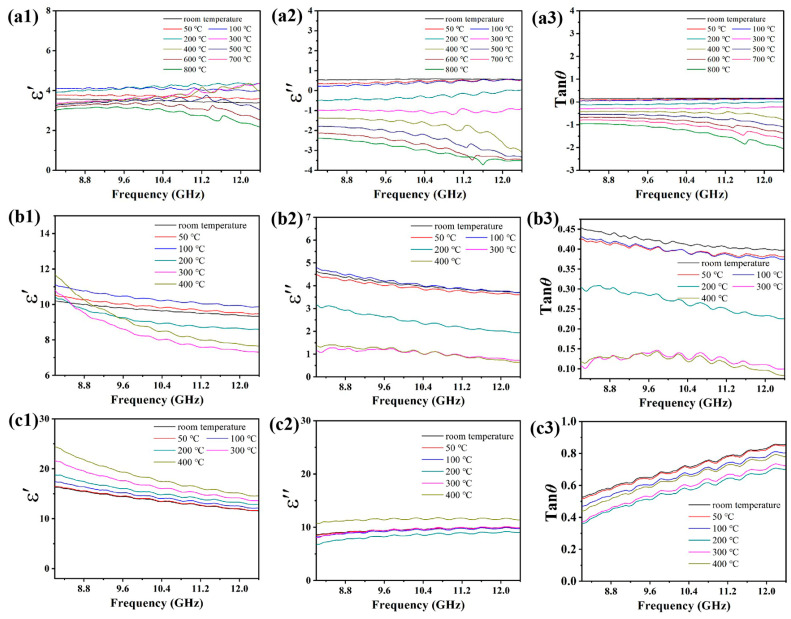
Dielectric properties of the composites with various C@TiC contents at different temperatures. (**a1**–**a3**) 10 wt.%; (**b1**–**b3**) 30 wt.%; and (**c1**–**c3**) 50 wt.%.

Figure 3 presents the real permittivity *ε*′, imaginary permittivity *ε*″, and dielectric loss tan *δ* of various C@TiC contents at elevated temperatures. Regardless of the test temperature, the *ε*′, *ε*″, and tan *δ* of all the composites demonstrate a gradual increase with increasing C@TiC content. For instance, at room temperature, the *ε*′, *ε*″, and tan δ of the composite with 10 wt.%C@TiC added are 3.38–3.59, 0.53–0.60, and 0.15–0.17, respectively, while the *ε*′, *ε*″, and tan *δ* of the composite with 50 wt.%C@TiC exhibit higher values of 11.53–16.35, 8.60–10.02, and 0.53–0.86, respectively. Furthermore, the *ε*′, *ε*″, and tan *δ* of the material exhibit the same variation with the changing C@TiC contents at higher temperature, which rose from 3.96 to 4.18, 0.22 to 0.57, and 0.05 to 0.14 at 10 wt.% C@TiC to 12.10–17.44, 8.19–9.92, and 0.47–0.81 at 50 wt.%C@TiC at 100 °C, respectively, in which the *ε*′ is highly associated with the polarization ability of the material [7,8,9]. On the one hand, the semiconducting TiC core and conductive C shell can not only generate electron displacement polarization under the influence of electromagnetic fields, but can also interface charge polarization at C/TiC interfaces. On the other hand, C@TiC/SiO_2_ interfaces can generate strong interface charge polarization. Additionally, significant dipolar and defect-induced polarization would be strongly built due to the low graphitic degree of the C-shell structure. All these factors including electron polarization, interface charge polarization, and dipolar- and defect-induced polarization can improve the *ε*′ of the composites. Apart from the *ε*′, the *ε*″ is mainly related to the conduction loss and polarization loss [10]. Undoubtedly, the increasing C@TiC absorbent will inevitably improve the conductivity of the material, i.e., enhance conduction loss. In addition, the enhanced polarization behaviors under the action of the electromagnetic field will also cause polarization loss, thereby increasing the *ε*″ of the material. Simultaneously, the tan *δ* derived from the ratio of *ε*″ to *ε*′ of the material also exhibits a similar variation trend as the *ε*″.

In addition to the C@TiC content, it can be discerned that the temperature exerts a great influence on the dielectric properties of composites with different C@TiC contents. As can be seen from Figure 1, as the temperature increases, the *ε*′ of the composites with 10 wt.% and 30 wt.%C@TiC initially rises and then drops, while the *ε*″ and tan *δ* undergo significant changes within the range of room temperature and 100 °C, indicating that the material has good stability within the temperature range. However, when the temperature exceeds 100 °C, the *ε*″ of the material decreases significantly, turning negative, especially for the composite with 10 wt.%C@TiC. This phenomenon primarily arises from the alteration of the C@TiC structure at elevated temperatures, which leads to anomalous variations in polarization mechanism. However, the C@TiC structure remains intact within the temperature range of room temperature to 400 °C, which means that the electrical conductivity and polarization capability originating from the C@TiC particle can still have a strong effect on both the real and imaginary permittivity. Therefore, the phenomenon discussed above can be mitigated to a certain extent for the composites with increased levels of C@TiC absorbent, providing a stable dielectric property at high temperatures. In particular, the 50 wt.%C@TiC composite exhibits a better high-temperature stability with no significant variation in the *ε*′, *ε*″, and tan *δ* within the range of room temperature to 400 °C.

### 3.3. Microwave Absorption Properties

**Figure 4:**
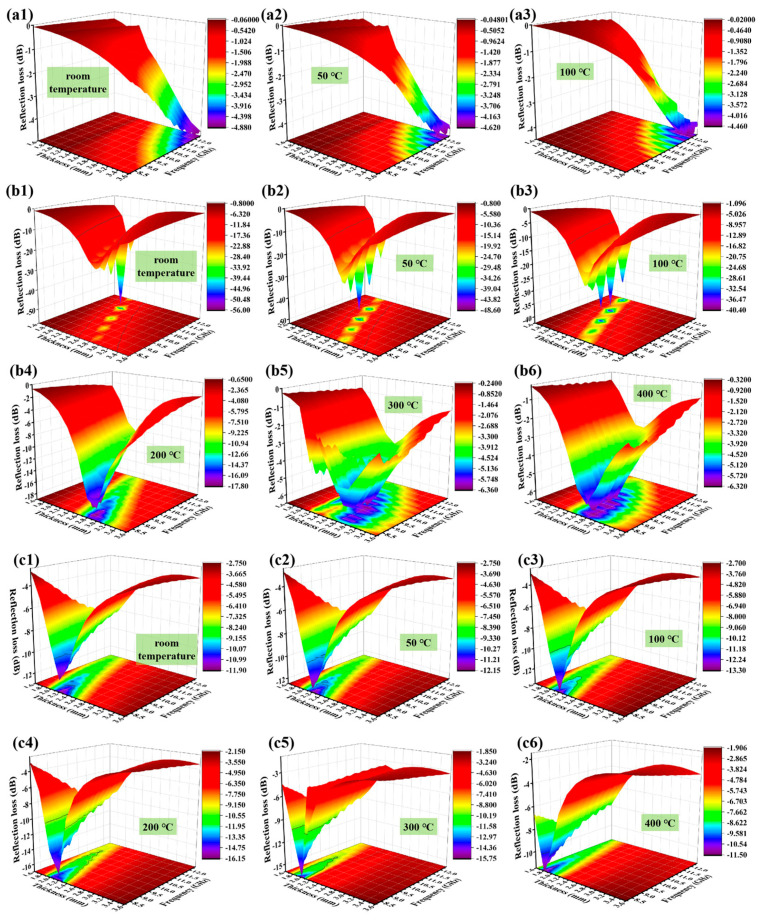
Reflection loss of the composite at different temperatures. (**a1**–**a3**) 10 wt.%C@TiC; (**b1**–**b6**) 30 wt.%C@TiC; and (**c1**–**c6**) 50 wt.%C@TiC.

The microwave absorption property of the material can be reflected by the reflection loss, which can be calculated using Equations (1) and (2) [11,12]:

(1) $$RL=20lg\left|\frac{Zin-Z0}{Zin+Z0}\right|$$

(2) $$Z_{\mathrm{in}}=Z_{0}\sqrt{\frac{\mu_{r}}{\varepsilon_{r}}}\tan h\left[j\frac{2\pi fd}{c}\sqrt{\mu_{r}\varepsilon_{r}}\right]$$

where *Z*_0_ and *Z*_in_ denote the free space impedance and the incident impedance of the material surface, respectively; *ε_r_* and *μ_r_* denote the complex permittivity and complex permeability of the material, respectively; *f* denotes the microwave frequency; c represents the light speed in a vacuum (3 × 10^8^ m·s^–1^); and d represents the material thickness. It is well acknowledged that the larger the absolute value of reflection loss, the stronger the microwave absorption property of the material [13]. Since the *ε*″ of the 10 wt.%C@TiC composite turns to a negative value when the temperature exceeds 100 °C, it can be deduced that the microwave absorption property of the material becomes significantly weaker at this point. Therefore, only the reflection loss within the range of room temperature to 100 °C and 1.6–3.6 mm thicknesses were calculated for the 10 wt.%C@TiC sample. The *RL* values of the composites with different C@TiC contents are presented in Figure 4. The *RL*_min_ of the 10 wt.%C@TiC composite at room temperature, 50 °C, and 100 °C is −4.63 dB (3.6 mm), −5.06 dB (3.6 mm), and −4.44 dB (3.2 mm), respectively, demonstrating a poor microwave absorption performance. This could be attributed to the low dielectric loss of the material, that is, the loss ability of the electromagnetic wave is weak in this case. In contrast, with the increasing C@TiC content to 30 wt.% and 50 wt.%, the microwave absorption performance of the material is remarkably improved. In particular, the 30 wt.%C@TiC composite presents excellent microwave absorption capacity with an *RL*_min_ of −55.87 dB (11.30 GHz, 2.2 mm), −48.49 dB (10.12 GHz, 2.4 mm), −40.36 dB (10.94 GHz, 2.2 mm), and −17.78 dB (8.59 GHz, 2.8 mm) at room temperature, 50 °C, 100 °C, and 200 °C, respectively, revealing that the material can absorb 99.999% of electromagnetic waves (*RL*_min_ ≤ −30 dB) within the range of room temperature to 100 °C. However, the microwave absorption performance of the 30 wt.% composite will be weakened with increasing temperature. When the temperature exceeds 200 °C, the absolute *RL*_min_ values of the 30 wt.% composite are less than 10 dB, which is also due to the decreasing dielectric loss. In contrast, as presented in Figure 4(c1–c6), the 50 wt.%C@TiC/SiO_2_ composite at room temperature to 400 °C can obtain a desirable *RL*_min_ value of −11.85 dB (8.22 GHz, 2.2 mm), −12.11 dB (8.21 GHz, 2.2 mm), −13.27 dB (8.2 GHz, 2.2 mm), −16.13 dB (8.2 GHz, 2.2 mm), −15.72 dB (8.2 GHz, 2.0 mm), and −11.48 dB (8.21 GHz, 1.8 mm) at room temperature, 50 °C, 100 °C, 200 °C, 300 °C, and 400 °C, respectively, meaning a 99% microwave absorption. Generally, based on the reflection loss results, the EM wave absorption performance of the 30 wt.% composite deteriorates as the temperature rises; this should be attributed to the increasing temperature exerting a more pronounced influence on the electromagnetic parameters. In contrast, the 50 wt.% composite demonstrates a better high-temperature stability and no significant change in view of electromagnetic parameters, which endows the composite with a more stable EM wave absorption performance with elevated temperature. Furthermore, it follows that increasing levels of C@TiC absorbent can enable the material to achieve a more effective absorption of microwaves at a smaller thickness compared with the 10 wt.%C@TiC composite. This is mainly because the increasing C@TiC content has a great regulation on the electromagnetic parameters of the material, according to the quarter-wavelength principle indicated by Equation (3) [14,15]:

*d*_m_ = *nλ*/4 = *n*c/(4*f*|*μ*_r_*ε*_r_|1/2) (*n* = 1, 3, 5…) (3)

where *d*_m_ refers to the material’s thickness, corresponding to the *RL* peak; the meaning of the other symbols is the same as above. The complex permittivity of the material is inversely proportional to the thickness corresponding to the *RL* peak. Therefore, as a result of the gradually increased complex permittivity with the increasing C@TiC content, the matching thickness of the reflection loss peak of the composites tends to a lower value.

Additionally, the effective absorption bandwidth (*RL* ≤ −10 dB) can also be significantly broadened by increasing the C@TiC content. As indicated by Table 1, the EABs of the 30 wt.%C@TiC composite are 3.29 GHz (2.4 mm), 3.19 GHz (2.4 mm), 3.22 GHz (2.4 mm), and 2.56 GHz (2.6 mm) at room temperature, 50 °C, 100 °C, and 200 °C, respectively. It is worth noting that through adjusting the material thickness within the range of 1.8–3.2 mm, the material can achieve an effective microwave absorption, covering the entire X-band at room temperature to 100 °C. In comparison, the 50 wt.%C@TiC composite exhibits a relatively narrow EAB at a thickness of 1.6–3.6 mm compared with the 30 wt.%C@TiC sample; however, its EAB at 200 °C and 300 °C can still reach satisfactory values of 2.21 GHz (2.4 mm) and 2.31 (2.4 mm), respectively.

As discussed above, the C@TiC addition can not only endow the composite with an enhanced microwave absorption performance, but also with an improved high-temperature microwave absorption performance. First, the improved microwave absorption performance of the composites can be attributed to the enhanced microwave loss capacity, in which the increased conduction loss with the increasing C@TiC content could enhance the loss capacity of materials to electromagnetic waves. Second, the polarization loss caused by electron displacement polarization, dipole polarization, defect-induced polarization, and interface charge polarization can contribute to electromagnetic wave loss. Third, the dispersed C@TiC particles in the SiO_2_ matrix can cause microwave scattering inside the material, and the microwave scattering ability will be stronger with increasing C@TiC content, thus improving the microwave absorption performance.

## 4. Conclusions

In this study, core–shell-structured C@TiC nanocomposites derived from Ti-based MOF precursors were utilized as high-efficiency microwave absorbers to prepare C@TiC/SiO₂ composites. The high-temperature microwave absorption performance of the composites was thoroughly investigated. The unique structure of C@TiC, combined with its intense conduction loss and multiple polarization mechanisms, contributes to the composites’ enhanced dielectric properties. As the content of C@TiC increases, both the permittivity and dielectric loss of the composites are significantly elevated, resulting in a superior microwave absorption performance at elevated temperatures. The composite with 30 wt.%C@TiC exhibits the strongest microwave absorption with an *RL*_min_ of −55.87 dB, −48.49 dB, and −40.36 dB at room temperature, 50 °C, and 100 °C, respectively. The 50 wt.%C@TiC composite with an *RL*_min_ below −10 dB at room temperature to 400 °C shows more stability at higher temperatures. This demonstrates that the C@TiC absorbent has a great potential to exhibit superior microwave absorption in high-temperature environments. Further studies relating to the long-term stability and durability of these composites under extended high-temperature and high-frequency microwave exposure will be important to evaluate their potential for practical use.

## Figures and Tables

**Table 1:** Summary of the *RL*_min_ and EAB values for the composites with various C@TiC absorber contents.

Temperature (°C)	30 wt.%C@TiC/SiO_2_ Composite		50 wt.%C@TiC/SiO_2_ Composite	
	*RL*_min_ (dB)/*d* (mm)	EAB (GHz)/*d* (mm)	*RL*_min_ (dB)/*d* (mm)	EAB (GHz)/*d* (mm)
Room temperature	−55.87/2.2	3.29/2.4	−11.85/2.2	0.83/2.4
50	−48.49/2.4	3.19/2.4	−12.11/2.2	0.85/2.4
100	−40.36/2.2	3.22/2.4	−13.27/2.2	1.55/2.4
200	−17.78/2.8	2.56/2.6	−16.13/2.2	2.21/2.4
300	−6.35/2.8	0	−15.72/2	2.31/2.4
400	−6.31/2.8	0	−11.48/1.8	0.76/2.4

## Data Availability

Data is contained within the article.
